# Supplementary material for: X-ray transient absorption reveals the 1Au (nπ*) state of pyrazine in electronic relaxation
Source: Nat Commun. 2021 Aug 18;12:5003. doi: 10.1038/s41467-021-25045-0 (PMC8373973; doi:10.1038/s41467-021-25045-0)
Supplement: Supplementary file 1 — Supplementary Information [file 41467_2021_25045_MOESM1_ESM.pdf]

Supplementary Information

X-ray transient absorption reveals the ^1A_u (nπ*) state of pyrazine in electronic relaxation

Valeriu Scutelnic[1], Shota Tsuru[2], Mátyás Pápai[2], Zheyue Yang[1], Michael Epshtein[1,3], Tian Xue[1], Eric Haugen[1,3], Yuki Kobayashi[1], Anna I. Krylov[4], Klaus B. Møller[2], Sonia Coriani[2], and Stephen R. Leone[1,3,5*]

[1]Department of Chemistry, University of California, Berkeley, CA 94720, USA

[2]DTU Chemistry, Technical University of Denmark, Kemitorvet Bldg 207, DK-2800, Kongens Lyngby, Denmark

[3]Chemical Sciences Division, Lawrence Berkeley National Laboratory, Berkeley, CA, 94720, USA

[4]Department of Chemistry, University of Southern California, Los Angeles, CA 90089, USA

[5]Department of Physics, University of California, Berkeley, CA 94720, USA

*Corresponding author: srl@berkeley.edu

Present addresses

Shota Tsuru: Ruhr-Universität, Bochum, Germany, 44780

Mátyás Pápai: Wigner Research Centre for Physics, P.O. Box 49, Budapest, Hungary, 1525

Michael Epshtein: Beer-Sheva, Israel 8471114

Zheyue Yang: No. 600 Cailun Road, Shanghai, China 201203

Yuki Kobayashi: Stanford PULSE Institute, SLAC National Accelerator Laboratory, Menlo Park, CA 94025, USA

Table of Contents

Supplementary Note 1: X-ray transient absorption spectra of pyrazine at long time delays3

Supplementary Note 2: Instrument response function (IRF) 4

Supplementary Note 3: Correcting for ground state bleach 5

Supplementary Note 4: Red-shift of X-ray $1s \rightarrow 2b_{3u}$ transition from the ^1A_u state 7

Supplementary Note 5: Geometrical effects on the calculated excited-state X-ray absorption spectra 9

Supplementary Note 6: Delay in population growth of ^1A_u state 14

Supplementary Note 7: Decay of 282.3 eV signal at longer times 15

Supplementary Note 8: Simulation of the hot ground-state spectrum at long delay 17

Supplementary Note 9: Ruling out internal conversion to ground state in the first 200 fs 19

Supplementary Note 10: Pump power dependence of the X-ray absorption spectra 20

Supplementary Note 11: Natural transition orbitals (NTOs) for valence excitations......... 23

Supplementary Note 12: NTOs for core-to-valence excitations....................................... 24

Supplementary References 28

Supplementary Note 1: X-ray transient absorption spectra of pyrazine at long time delays

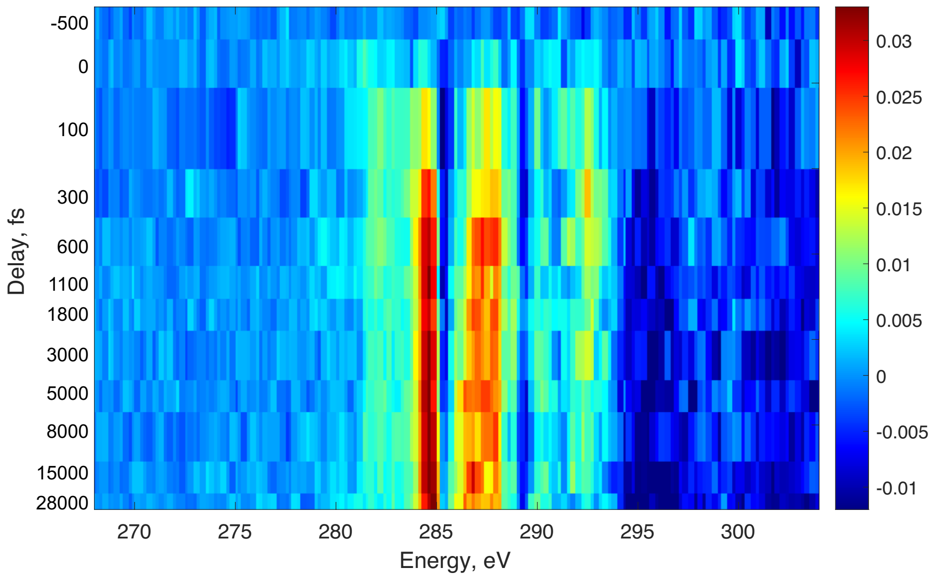

Supplementary Figure 1. **Dynamics at long delays.** Heat map showing X-ray absorption spectra of pyrazine measured at long delays (up to 28 ps). A logarithmic scale is used for the time delay axis.

Supplementary Note 2: Instrument response function (IRF)

The response function of the apparatus is determined using the AC-Stark shift of the core-excited Rydberg states in argon under the same UV-pump X-ray probe configuration (Supplementary Figure 2a).[1] The highest magnitude of the signal in Supplementary Figure 2b corresponds to the temporal overlap of the pump and probe and is defined as time zero.

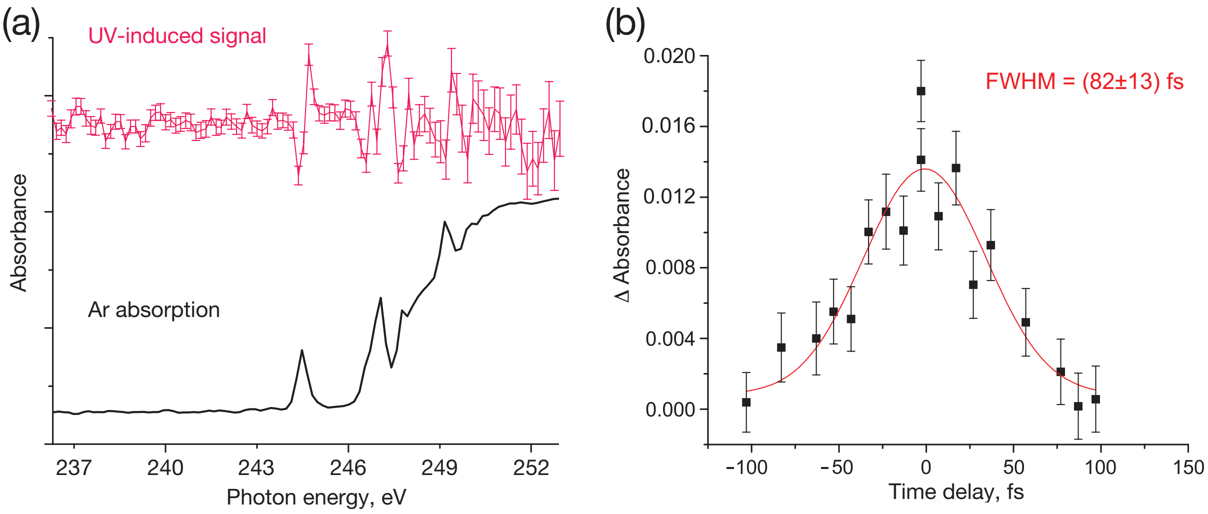

Supplementary Figure 2. **Measurement of instrument response function.** (a) Black: argon X-ray absorption spectrum, red: AC-Stark shift induced by the UV pulse at full overlap with the X-ray pulse. (b) The delay dependence of the signal at 244.6 eV, corresponding to the argon transition $^2p_{3/2}\rightarrow 4s$. Error bars are one standard deviation of 128 measurements.

Supplementary Note 3: Correcting for ground state bleach

X-ray transient absorption spectra are composed of the positive absorption signals of excited states and a negative absorption signal from the ground state, as its population is depleted by the UV pump. In the experimental conditions of ~30±6 mJ·cm^{-2} pump fluence (2.5×10^{11} W·cm^{-2} intensity) and with an absorption cross section of 7 Mb at the pump wavelength of 267 nm,[2] up to 25% of the pyrazine molecules are excited. This estimate relies on the measurement precision of the UV pulse energy, obtained with an uncertainty of <1%, and spot size, estimated to within 20%. These uncertainties propagate to an upper limit of variability in the determined excitation fraction of (25±5) %. In Supplementary Figure 3, the filled spectra show that the principal change in spectra with 20% and 30% add-back is most clearly revealed in the alteration of the intensity of the 285.4 eV band, which is not used in the assignment of $^1B_{2u}$, $^1B_{3u}$ and ^1A_u states and does not change any of the conclusion. An UV excitation of 23% is found to be optimal for removing the discontinuities (highlighted with vertical dashed lines in Supplementary Figure 3) caused by the ground state bleach. This number is very close to the 25% estimate from the pulse energy, beam volume, and cross section.

At delays >100 fs, the pump and probe pulses are well separated in time and the estimated (25±5) % of the excited molecules is subsequently probed. When the UV pump and X-ray probe pulses overlap in time (at -20 fs and 20 fs delays in Figure 5), the fraction of excited and probed molecules $p_{\text{excited\&probed}}$ varies depending on the pump-probe delay t, due to the temporal overlap of the UV pulse with the X-ray probe pulse, initially giving a much smaller fraction and eventually reaching the full excited population in time:

$$p_{\text{excited\&probed}}(t) = p_{\text{exc}} \cdot \int_{-\infty}^{t} (f_{\text{UV}} * f_{\text{Xray}})(\tau) d\tau = p_{\text{exc}} \cdot \int_{-\infty}^{t} \text{IRF}(\tau) d\tau = p_{\text{exc}} \cdot \frac{1+\text{erf}\left(\frac{t}{\sqrt{2}\cdot\sigma_{\text{IRF}}}\right)}{2},$$

where p_{exc} is the percent of molecules initially excited by the UV pulse (23% used in the add-back spectrum), f_{UV} and f_{Xray} are the temporal profiles of the UV and X-ray pulses (approximately gaussians), and IRF represents the instrument response function with a standard deviation $\sigma_{\mathrm{IRF}} = (35 \pm 6) fs$ (see Supplementary Figure 2). Thus, $p_{\mathrm{excited\&probed}}(-20\ fs) = 7\%$ and $p_{\mathrm{excited\&probed}}(20\ fs) = 15\%$ are applied in Figure 5.

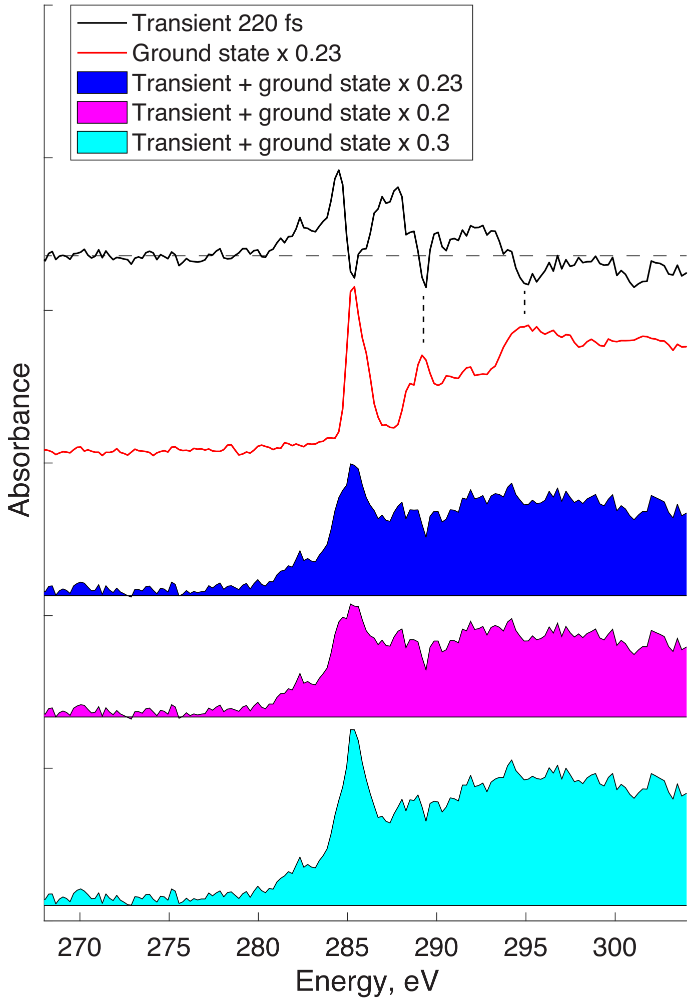

Supplementary Figure 3. **Correction for the ground state bleach of the transient spectrum acquired at 220 fs.** Black: differential X-ray spectrum acquired at 220 fs; dashed line is reference zero line. Red: Scaled ground state. Filled blue: the sum of the transient spectrum and 23% of the ground state. Filled purple: the sum of the transient spectrum and 20% of ground state. Filled cyan: the sum of the transient spectrum and 30% of ground state.

Supplementary Note 4: Red-shift of X-ray 1s→2b_{3u} transition from the ^1A_u state

A characteristic red shift of the 1s→2b_{3u} transition from the ^1A_u state compared to the same core-valence transition from the ground S_0 state is observed in the experiment. The 6a_g→1a_u valence excitation to the ^1A_u state leads to electron charge density shift from nitrogen to carbon atoms, because the 1a_u orbital is localized on the four carbon centers (see Supplementary Figure 4). For this reason, the red shift of the X-ray transition from the ^1A_u state can be rationalized by a reduced repulsion between the electrons occupying the 1a_u and 1s orbitals after core excitation into the 2b_{3u} orbital, which has electron density on the nitrogen atoms. Apart from this, the core-excited state is stabilized by more efficient Coulomb attraction between the carbon nuclear charge and the electron occupying the 1a_u orbital.

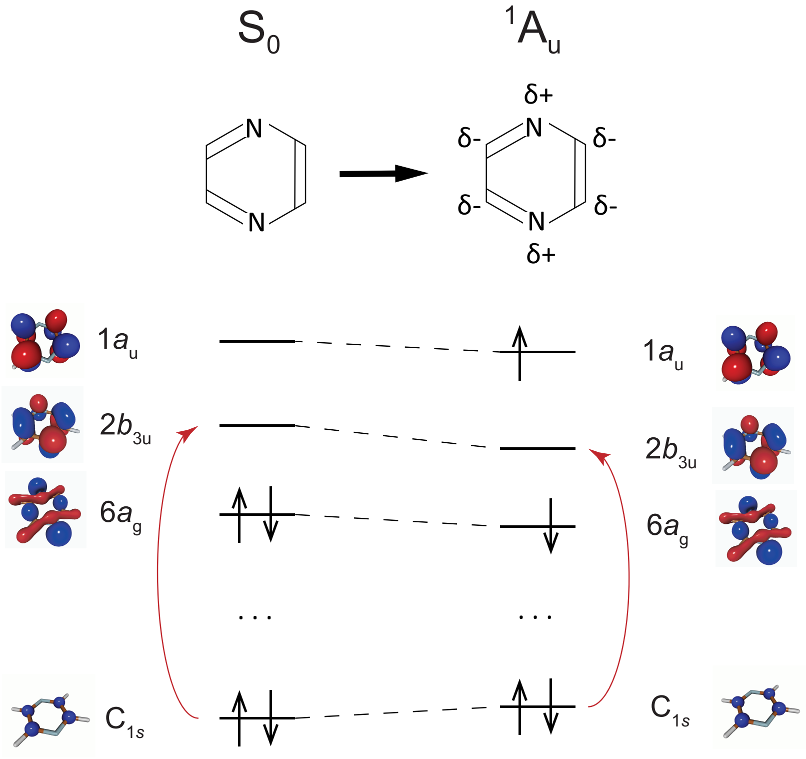

Supplementary Figure 4. **Electron transfer from nitrogen to carbon in the ^1A_u state and the shift of core-to-valence transition.** Top: Valence excitation to the

^1A_u state leads to electron charge shift to the carbon atoms. Bottom: Schematic representation of the dominant core-valence transitions from the ground S_0 and ^1A_u states (for spin complete representation, see Supplementary Table 5).

Supplementary Note 5: Geometrical effects on the calculated excited-state X-ray absorption spectra

The low-energy peaks in the experimentally extracted excited-state spectra in Figures 5a and 5b of the main article are located at 281.5 eV (−20 fs time delay) and 282.3 eV (+20 fs time delay). Based on the spectra calculated at the Franck-Condon (FC) geometry, the spectral signatures are assigned to the $^1B_{2u}$ (281.5 eV, $1s\rightarrow 1b_{1g} - \pi$) and $^1B_{3u}$ (282.3 eV, $1s\rightarrow 6a_g - n$) states (see Supplementary Figures 5, 6 and Supplementary Tables 3, 4). However, the FC-calculated peaks are centered at 280.7 eV ($^1B_{2u}$) and 281.8 eV ($^1B_{3u}$), leading to 0.8 eV and 0.5 eV discrepancies with the experimental values, respectively. Here we show that this mismatch decreases by accounting for geometrical effects. Supplementary Figures 5 and 6 show the trajectory-averaged $^1B_{2u}$ (18 structures) and $^1B_{3u}$ spectra (28 structures) at −20 fs and +20 fs, respectively; -20 fs in the experiment corresponds to 140 fs in the surface hopping (SH) simulation, because the 0 fs is set to the pump maximum in the experiment. We have selected the structures with minimal valence electronic mixing, because the $^1B_{2u}$/$^1B_{3u}$/^1A_u mixing would interfere with identifying the geometrical effect. Such mixing is inevitable in the case of the 220 fs (Supplementary Figure 7) time delay due to strong geometry distortion. As seen in Figure S5, the low-energy peaks are shifted to 281.2 eV ($^1B_{2u}$) and 282.0 eV ($^1B_{3u}$), decreasing the deviation from experiment to 0.3 eV in both cases (which is the same as the resolution of our X-ray spectrometer). Importantly, the separation of the two peaks decreases from 1.1 eV (at FC geometry) to 0.8 eV, the latter being in perfect agreement with the experimental value. We have found that the peak positions are converged with respect to the number of excited-state structures utilized in the calculations. We have also calculated the averaged spectra for the ground-state Wigner distribution, resulting in 281.0 eV ($^1B_{2u}$) and 281.9 eV ($^1B_{3u}$) peak positions, as well as that of the ^1A_u state, resulting in its 281.4 eV peak position. This shows that the geometric effect

has a significant dynamic character. Note that in the simulated dynamics spectrum of Figure 6b in the main article, which is the same as the blue curve in Figure S7, geometrical effects are accounted for and the position of the low-energy peak is indeed in good agreement with the experimental one. Finally, in Figure S8, we illustrate the convergence of the simulated dynamics (trajectory-averaged) spectrum with respect to the number of utilized trajectories. This clearly shows that the simulated X-ray absorption spectrum utilizing 59 trajectories is converged.

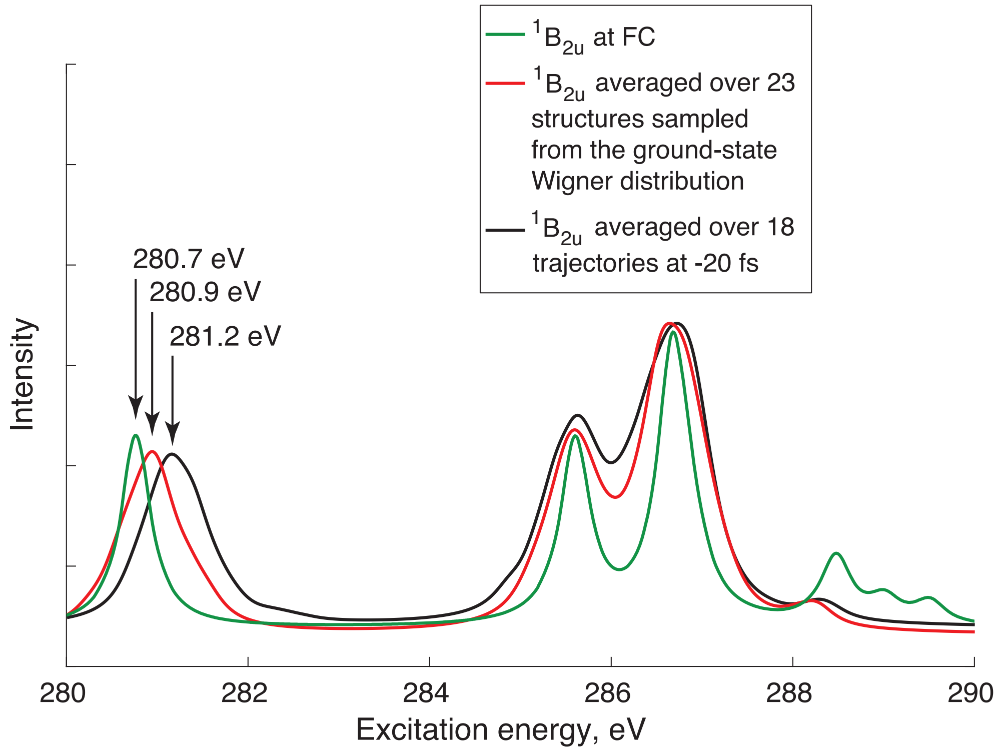

Supplementary Figure 5. **Computed absorption spectra from $^1B_{2u}$ state**. Green: Computed $^1B_{2u}$ spectrum at the Franck-Condon geometry. Red: Average of 23 X-ray spectra for the $^1B_{2u}$ state obtained from the ground-state Wigner distribution. Black: Trajectory-averaged $^1B_{2u}$ X-ray spectrum simulated at −20 fs time delay, calculated using 18 excited-state structures. The excitation energies have been shifted by 10.7 eV.

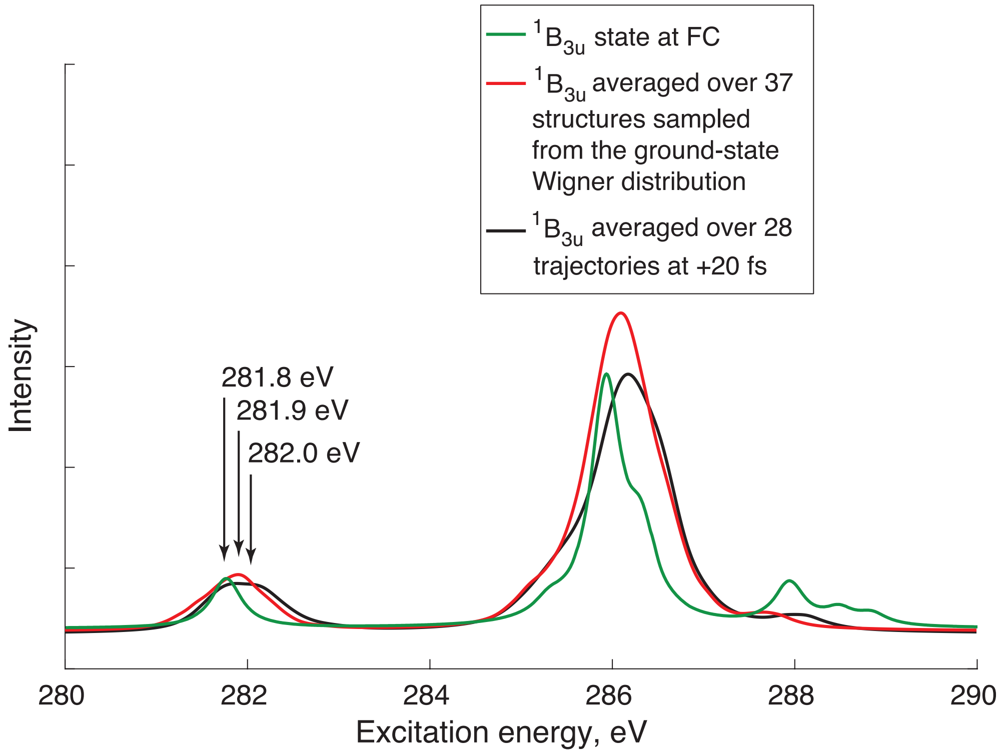

Supplementary Figure 6. **Computed absorption spectra from $^1B_{3u}$ state**. Green: Computed $^1B_{3u}$ spectrum at the Franck-Condon geometry. Red: Average of 37 X-ray spectra for the $^1B_{3u}$ state obtained from the ground-state Wigner distribution. Black: Trajectory-averaged $^1B_{3u}$ X-ray spectrum simulated at 20 fs time delay, calculated using 28 excited-state structures. The excitation energies have been shifted by 10.7 eV.

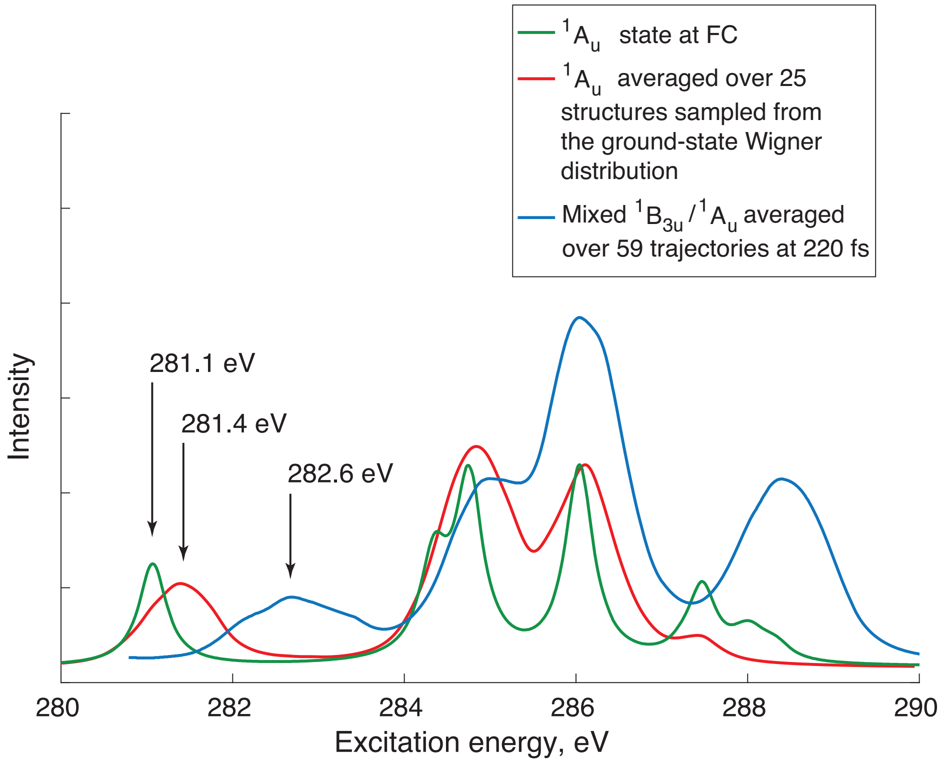

Supplementary Figure 7. **Computed absorption spectra from ^1A_u state**. Green: Computed ^1A_u spectrum at the Franck-Condon geometry. Red: Average of 25 X-ray spectra for the ^1A_u state obtained from the ground-state Wigner distribution. Black: Trajectory-averaged X-ray spectrum with mixed $^1B_{3u}/^1A_u$ character simulated at 220 fs time delay, calculated using 59 excited-state structures. The excitation energies have been shifted by 10.7 eV.

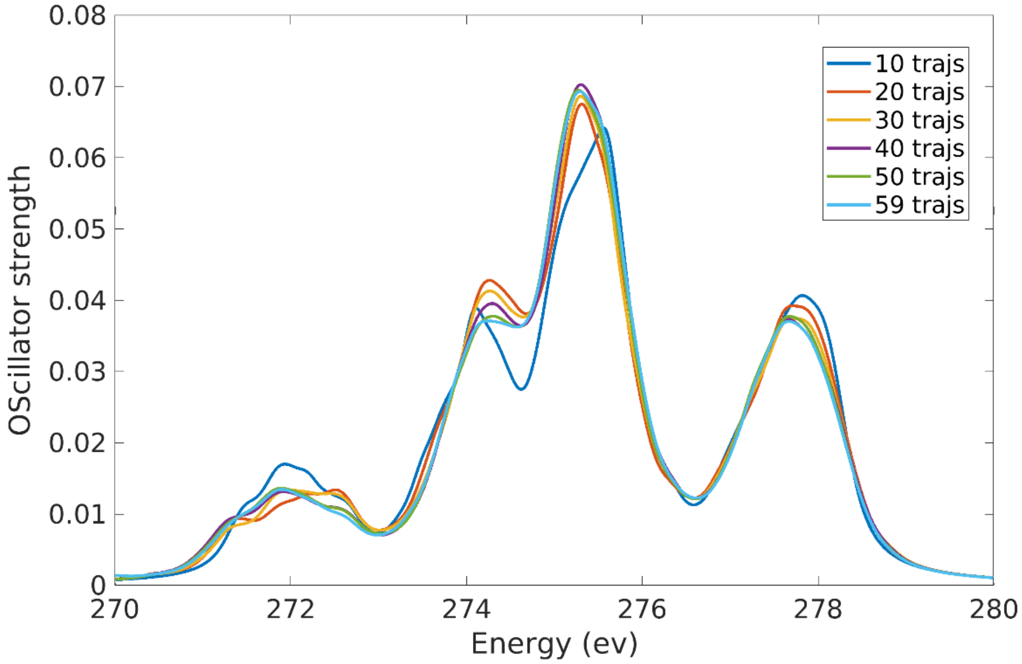

Supplementary Figure 8. **Test of convergence of the simulated dynamics.** Trajectory-averaged spectrum at 220 fs constructed with 10, 20, 30, 40, 50 and 59 trajectories of the 59 trajectories of the SH simulation.

Supplementary Note 6: Delay in population growth of ^1A_u state

The experimentally observed ~200 fs slower population growth of the ^1A_u state compared with the $^1B_{3u}$ state was observed in the nuclear dynamics simulation of Sala *et al.*,[3] while in the simulation of Tsuru *et al.*[4] the population of ^1A_u was not delayed from the $^1B_{3u}$ state. Sala *et al.*, adjusted empirically the vertical excitation energies of the $^1B_{3u}$, ^1A_u, and $^1B_{2u}$ states for consistency with the experimental UV absorption spectrum.[3] In Ref. 4 instead, the vertical excitation energies were used directly from the XMCQDPT2 calculations.[5] The empirical adjustment made the potential energy minimum of the ^1A_u state higher than that of the $^1B_{3u}$ state. Therefore, this adjustment may be responsible for the slower flow of population into ^1A_u, as observed in this experiment. As seen in Table 3 of Ref. 5, the energy ordering of the ^1A_u and $^1B_{2u}$ states depends on the level of theory to a large extent, whereas the experimental determination of the state ordering is hindered by the dipole selection rules. As the delayed increase of the ^1A_u state has been reproduced in Sala's population dynamics, where the ^1A_u state is only 0.10 eV below the $^1B_{2u}$ state vertically,[3] the present work suggests that the vertical excitation energies of ^1A_u and $^1B_{2u}$ are similar.

Supplementary Note 7: Decay of 282.3 eV signal at longer times

At longer times the absorption at 282.3 eV, which corresponds to the $1s \rightarrow 6a_g$ (n) transition from both $^1B_{3u}$ and ^1A_u states, shows a gradual decrease (Supplementary Figure 9). The poor signal-to-noise prevents us from establishing the decay constant of this absorption band, nonetheless the data points suggest a decay with 15–20 ps time constant, consistent with the internal conversion to the ground state.[6,7] The nonzero signal after 100 ps is an indication of other excited states, which could possibly be spin-triplet states (Supplementary Figure 10).

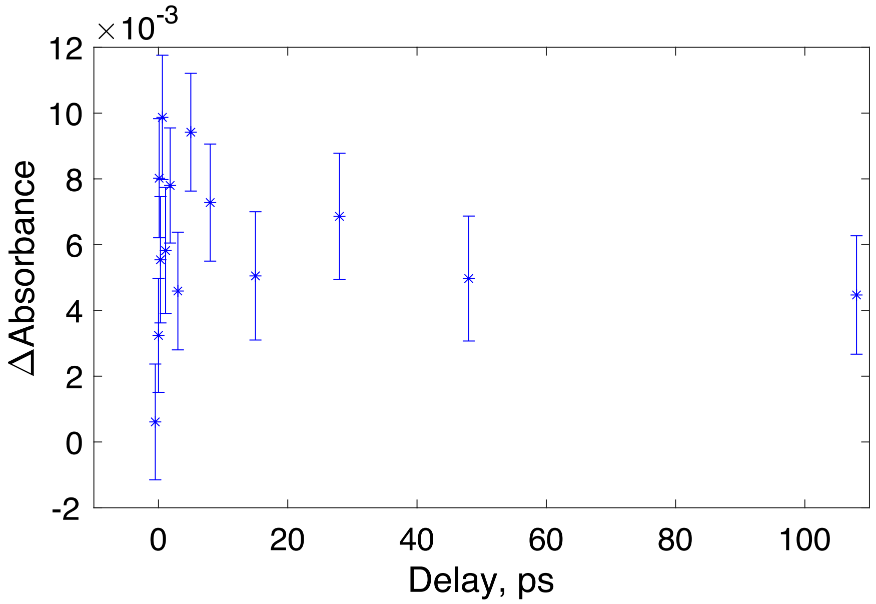

Supplementary Figure 9. **Decay to the ground electronic state.** Decay of the signal at 282.3 eV. Error bars represent one standard deviation of 128 measurements.

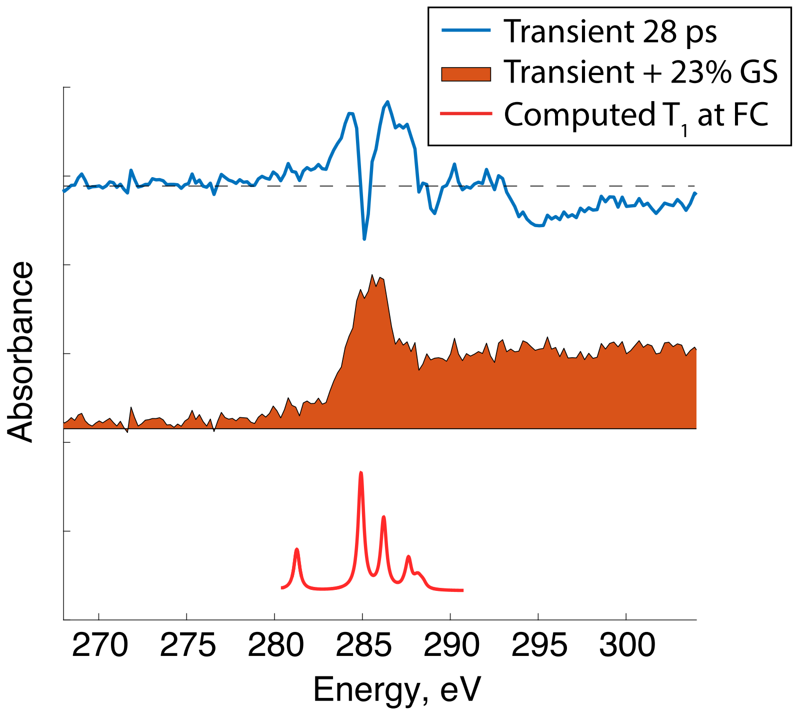

Supplementary Figure 10. **Potential detection of the triplet state at 28 ps.** Blue: experimental differential spectrum at 28 ps. Brown filled trace: differential spectrum corrected for ground state bleach by adding 23% of ground state. Red: computed triplet state spectrum at Franck-Condon geometry.

Supplementary Note 8: Simulation of the hot ground-state spectrum at long delay

The electronic wave functions are computed in the diagonal (spin-adiabatic) representation, i.e., by diagonalization of the non-relativistic Hamiltonian[8] plus the spin-orbit coupling (SOC) perturbation. 63 pairs of initial momenta and geometries are generated with a stochastic procedure employing the excitation energies and oscillator strengths at the geometries sampled from the ground-state Wigner distribution.[9] Aiming at obtaining at least one trajectory reaching the ground state, these calculations are carried out for 18 of the 63 pairs. One trajectory has undergone two non-adiabatic transitions: one from a singlet to a triplet state at 335.5 fs and one at 1962.0 fs into the ground state. Since conical intersections with the ground state cannot be accurately described at the DFT/TDDFT level, an irreversible hop to the ground state is forced when the energy gap became less than 0.3 eV, using the option `force_hop_to_gs` in SHARC. From 1962.0 to 4862.0 fs, 30 geometries have been sampled along this trajectory every 100 fs and a spectrum has been computed for each geometry. Finally, the X-ray absorption spectrum of the vibrationally hot ground state has been calculated as the average of these 30 spectra (Supplementary Figure 11).

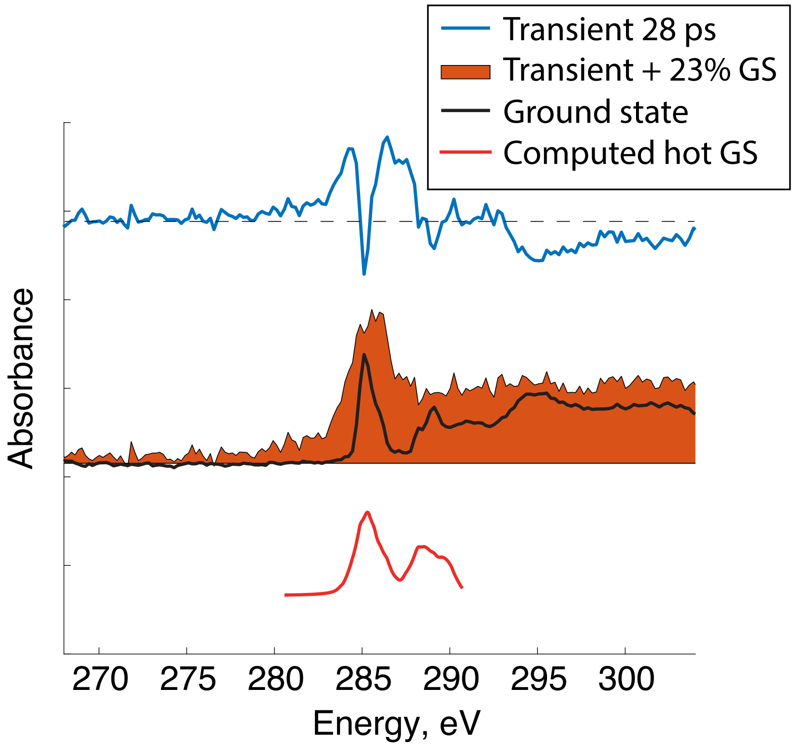

Supplementary Figure 11. **Vibrationally hot ground state at 28 ps.** Experimental differential spectrum acquired at 28 ps (blue), differential spectrum corrected for ground state bleach (brown area), experimental ground state absorption spectrum (black), computed vibrationally hot ground state produced by radiationless decay (red).

Supplementary Note 9: Ruling out internal conversion to ground state in the first 200 fs

The generation of vibrationally hot ground state within 200 fs is unlikely for a few reasons. First, we rule out the possibility of decay via the $^1B_{2u}/^1A_g$ conical intersection. According to the experimental study by Stener *et al.*,[10] the band maximum and the 0–0 transition of the $^1B_{2u}$ state of pyrazine lie at 4.81 eV and 4.69 eV, respectively. Since pyrazine is pumped with a 267 nm (4.64 eV, 0.04 eV bandwidth) pulse in the experiment, the nuclear wave packet on the $^1B_{2u}$ state surface has a small excess kinetic energy. The excess energy is not sufficient to reach the conical intersection between the $^1B_{2u}$ state and the ground state, which is located ~0.7 eV higher than the potential energy minimum of the $^1B_{2u}$ state (in analogy with the prefulvene pathway observed in benzene[11]). Second, we rule out the possibility of decay via the $^1B_{3u}/^1A_g$ or the $^1A_u/^1A_g$ conical intersection. The $^1B_{2u}/^1B_{3u}$ and the $^1B_{3u}/^1A_g$ (or $^1A_u/^1A_g$) conical intersections lie 2.1 eV and 1.7 eV above the potential energy minimum of $^1B_{3u}$ (or ^1A_u) at the CASSCF(6,6)/6-31G* level of theory.[12] Thus, the nuclear wave packet generated on the $^1B_{3u}$ (or ^1A_u) has excess kinetic energy sufficient to reach the $^1B_{3u}/^1A_g$ (or $^1A_u/^1A_g$) conical intersection. However, such a decay would typically occur on picosecond timescales when much of the excess kinetic energy is in the reaction coordinate.

Supplementary Note 10: Pump power dependence of the X-ray absorption spectra

As the absorption cross section of pyrazine is very high (7 Mb at 267 nm)[2] the one-photon excitation is more efficient than the two-photon ionization. Moreover, if a two-photon process would occur, the formed ion would be in its ground state with nearly zero internal energy (pyrazine ionization potential 9.0 eV,[13] two 267 nm photons carry about 9.3 eV) and would not be able to undergo dynamics on the 200 fs timescales, thus the ionic features would appear on the duration of the IRF as in the case of the benzene radical cation.[14] Instead, the 284.5 eV band assigned to the ^1A_u state has a time constant more than twice as long as the IRF. To verify that the UV excitation in pyrazine is indeed due to one photon, the X-ray spectra have been recorded at different UV powers (Supplementary Figure 12). Lower pump power results in a weaker signal, while the overall shape of the spectrum does not change significantly, in particular the ratio of the bands peaking at 282.3 and 284.5 eV is nearly the same.

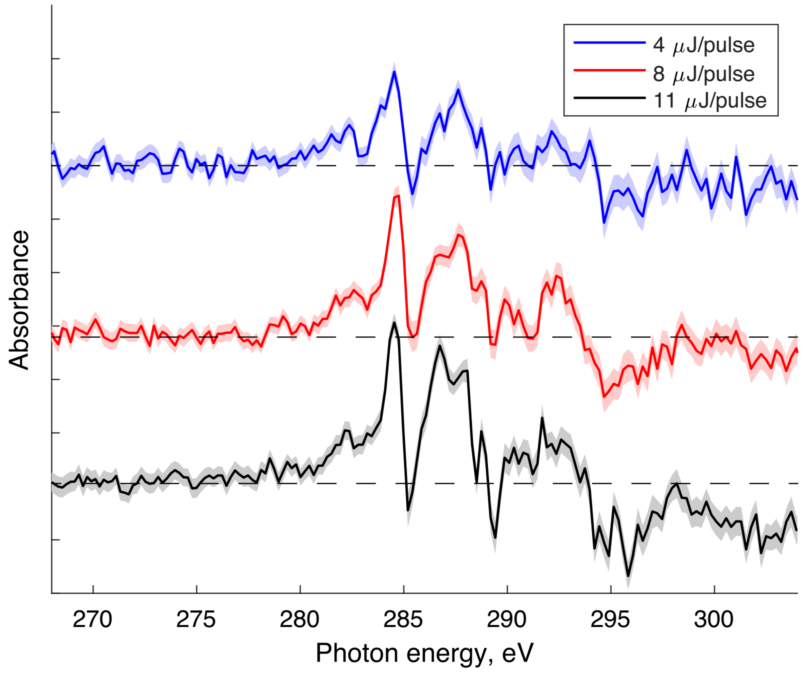

Supplementary Figure 12. **Pump power dependence of X-ray absorption spectra.** Differential absorption spectra acquired at 3 ps delay. Shaded area represents one standard deviation of 128 measurements.

According to Lambert-Beer law, the X-ray attenuation of the UV-excited species is expressed as:

$$\frac{I_{\mathrm{Xray,UV}}}{I_{\mathrm{Xray,0}}} = e^{-\sigma_{\mathrm{Xray}} \cdot L \cdot n_{\mathrm{UVexcited}}}, \quad (1)$$

where $I_{\mathrm{Xray,UV}}$ and $I_{\mathrm{Xray,0}}$ are the intensity of transmitted X-rays with and without UV, σ_{Xray} is the X-ray absorption cross section of the UV excited species, L is the cell length and $n_{\mathrm{UVexcited}}$ is the number density of the UV excited pyrazine molecules in the probed volume. At 284.5 eV the X-ray absorption of the ground state is negligible. The UV photons excite part of pyrazine molecules according to the formula:

$$1 - \frac{N_{\mathrm{UVexcited}}}{N_{\mathrm{pyrazine}}} = e^{-\sigma_{\mathrm{UV}} \cdot F_{\mathrm{UV}}}, \quad (2)$$

where $N_{\mathrm{UVexcited}}$ is the number of pyrazine molecules excited by the UV laser, N_{pyrazine} is the total number of molecules, σ_{UV} is the absorption cross section of pyrazine at 267 nm and F_{UV} is the pump fluence. Combining equations (1) and (2) results in:

$$\Delta A = \log_{10} e \cdot \sigma_{\mathrm{Xray}} \cdot L \cdot n_{\mathrm{pyrazine}} \cdot (1 - e^{-\sigma_{\mathrm{UV}} \cdot F_{\mathrm{UV}}}) = c1 * (1 - e^{-c2 \cdot P_{\mathrm{UV}}}), \quad (3)$$

where ΔA is the measured differential absorbance, *c1* and *c2* are constants and P_{UV} is UV power.

The experimentally determined UV-pump power dependence of the X-ray absorption signal is displayed in Supplementary Figure 13. The data points fit well to the equation (3) derived from Lambert-Beer law, confirming the absorption of one UV photon.

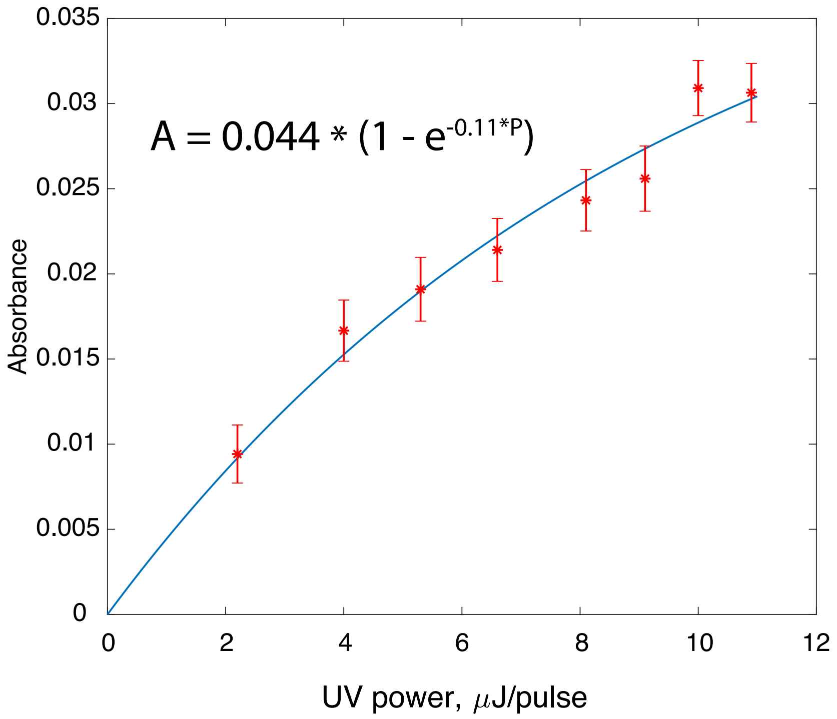

Figure S13. **UV-pump power dependence of the X-ray absorption signal at 284.5 eV.** In the fitting equation A is absorbance and P is UV power in μJ/pulse. Error bars are one standard deviation of 128 measurements.

Supplementary Note 11: Natural transition orbitals (NTOs) for valence excitations

Pyrazine is easily excited to the $^1B_{2u}$ ($\pi\pi^*$) state by 267 nm photons due to a high oscillator strength for this transition (~0.1). Transition from the ground state to ^1A_u has zero oscillator strength and is inaccessible by UV excitation. Both $^1B_{3u}$ and ^1A_u are nπ^* states with the hole orbital being 6a_g, while the particle orbital is 2b_{3u} and 1a_u, respectively.

Supplementary Table 1. NTOs of the valence excitations from the ground state computed at B3LYP/6-311++G** level of theory. Each hole/particle NTO closely resembles the canonical molecular orbital whose index is given in parenthesis.

State	Excitation energy (eV)	Oscillator strength	Hole		σ_K^2	Particle	
$^1B_{3u}$(nπ^*)	3.94	0.0056	a_g (6a_g)	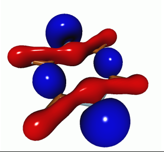	1.00	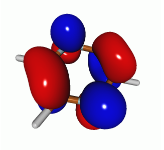	b_{3u} (2b_{3u})
^1A_u(nπ^*)	4.62	0	a_g (6a_g)	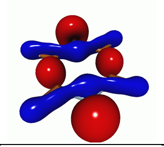	1.00	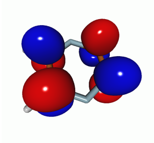	a_u (1a_u)
$^1B_{2u}$($\pi\pi^*$)	5.44	0.0968	b_{1g} (1b_{1g})	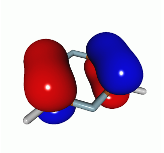	0.88	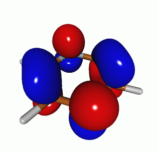	b_{3u} (2b_{3u})
			b_{2g} (1b_{2g})	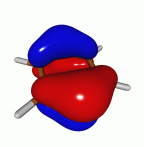	0.12	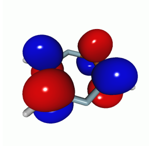	a_u (1a_u)

Supplementary Note 12: NTOs for core-to-valence excitations

Supplementary Table 2. NTOs of the lowest-lying C_{1s} excitations from the ground state to each unoccupied valence orbital. Excitation energies have been shifted by +10.7 eV. Each hole/particle NTO closely resembles the canonical molecular orbital whose index is given in parenthesis.

Excitation energy (eV)	Oscillator strength	Hole		σ_K^2	Particle	
285.4	0.0973	a_g ($2a_g$)	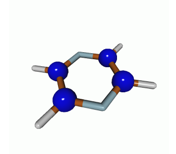	0.86	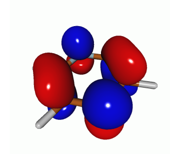	b_{3u} ($2b_{3u}$)
		b_{3g} ($1b_{3g}$)	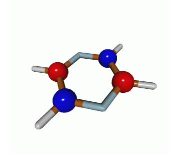	0.12	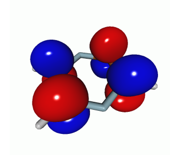	a_u ($1a_u$)
286.1	0.0637	b_{3g} ($1b_{3g}$)	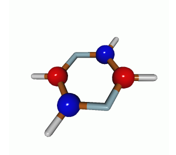	0.86	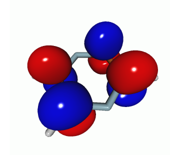	a_u ($1a_u$)
		a_g ($2a_g$)	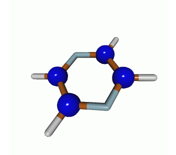	0.13	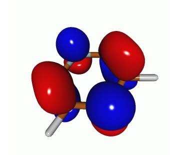	b_{3u} ($2b_{3u}$)
287.9	0.0048	b_{1u} ($2b_{1u}$)	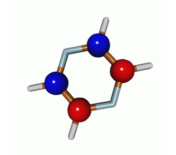	0.84	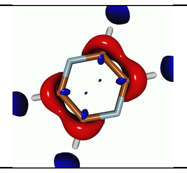	a_g ($10a_g$)
		b_{3g} ($1b_{3g}$)	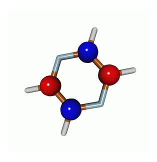	0.15	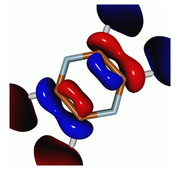	b_{2u} ($7b_{2u}$)
287.9	0.0127	b_{2u} ($1b_{2u}$)	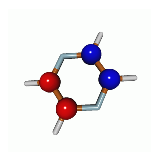	0.83	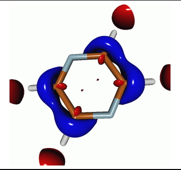	a_g ($10a_g$)
		a_g ($2a_g$)	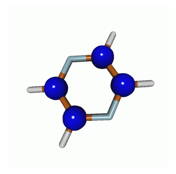	0.15	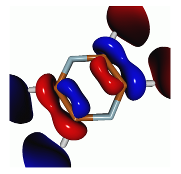	b_{2u} ($7b_{2u}$)

289.5	0.0171	a_g ($2a_g$)	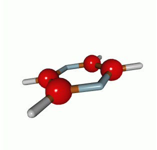	0.95	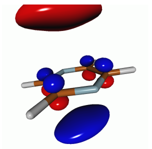	b_{3u} ($6b_{3u}$)

Supplementary Table 3. NTOs of the lowest-lying C_{1s} excitations from the $^1B_{2u}$ state at the FC geometry. Excitation energies have been shifted by +10.7 eV.

Excitation energy (eV)	Oscillator strength	α			β		
		Hole	σ_K^2	Particle	Hole	σ_K^2	Particle
280.7	0.0517	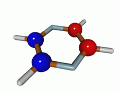	0.98	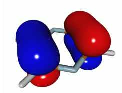			
285.5	0.0487				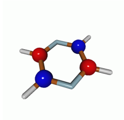	0.82	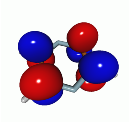
286.6	0.0701	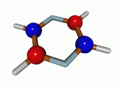	0.83	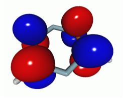			
286.8	0.0143				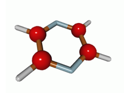	0.88	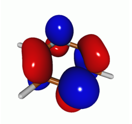
288.4	0.0126	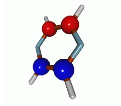	0.30	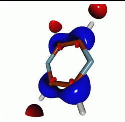	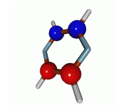	0.52	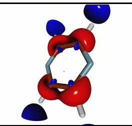
					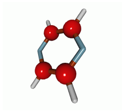	0.11	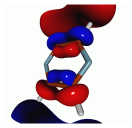

Supplementary Table 4. NTOs of the lowest-lying C_{1s} excitations from the $^1B_{3u}$ state at the FC geometry. Excitation energies have been shifted by +10.7 eV.

<table>
<tr><th rowspan="2">Excitation energy (eV)</th><th rowspan="2">Oscillator strength</th><th colspan="3">α</th><th colspan="3">β</th></tr>
<tr><th>Hole</th><th>σ_K^2</th><th>Particle</th><th>Hole</th><th>σ_K^2</th><th>Particle</th></tr>
<tr><td>281.8</td><td>0.0201</td><td>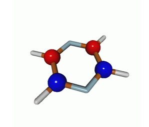</td><td>1.00</td><td>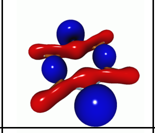</td><td colspan="3"></td></tr>
<tr><td rowspan="2">285.3</td><td rowspan="2">0.0071</td><td>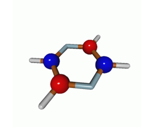</td><td>0.28</td><td>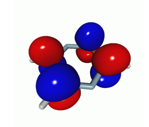</td><td>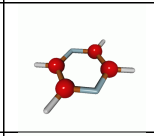</td><td>0.36</td><td>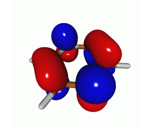</td></tr>
<tr><td colspan="3"></td><td>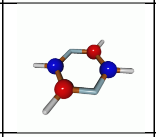</td><td>0.31</td><td>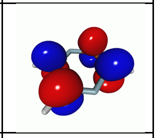</td></tr>
<tr><td>286.0</td><td>0.1029</td><td>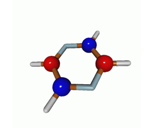</td><td>0.61</td><td>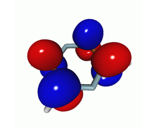</td><td>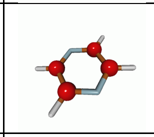</td><td>0.34</td><td>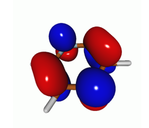</td></tr>
<tr><td rowspan="2">286.3</td><td rowspan="2">0.0337</td><td rowspan="2" colspan="3"></td><td>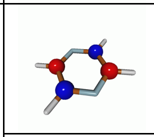</td><td>0.64</td><td>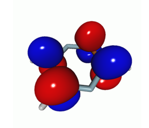</td></tr>
<tr><td>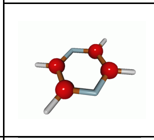</td><td>0.29</td><td>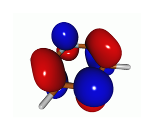</td></tr>
<tr><td>288.0</td><td>0.0133</td><td>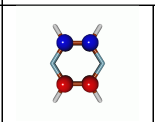</td><td>0.45</td><td>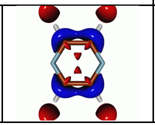</td><td>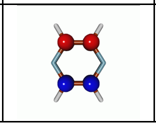</td><td>0.38</td><td>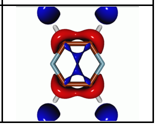</td></tr>
</table>

Supplementary Table 5. NTOs of the lowest-lying C_{1s} excitations from the $^{1}A_u$ state at the FC geometry. Excitation energies have been shifted by +10.7 eV.

Excitation energy (eV)	Oscillator strength	α			β		
		Hole	σ_K^2	Particle	Hole	σ_K^2	Particle
281.1	0.0214	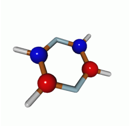	1.00	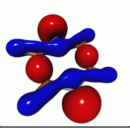			
284.4	0.0210	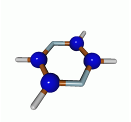	0.12	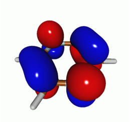	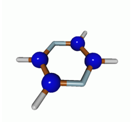	0.79	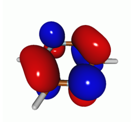
284.8	0.0391	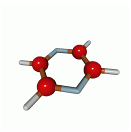	0.85	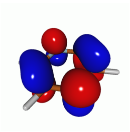	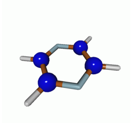	0.14	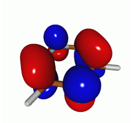
286.1	0.0428				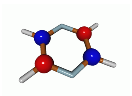	0.88	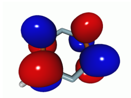
287.6	0.0119	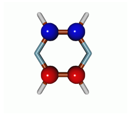	0.61	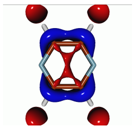	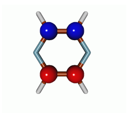	0.23	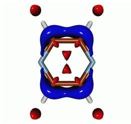
		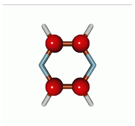	0.11	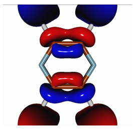			

Supplementary References

(1) Bhattacherjee, A.; Pemmaraju, C. D.; Schnorr, K.; Attar, A. R.; Leone, S. R. Ultrafast Intersystem Crossing in Acetylacetone via Femtosecond X-Ray Transient Absorption at the Carbon K-Edge. *J. Am. Chem. Soc.* **2017**, *139* (46), 16576–16583. https://doi.org/10.1021/jacs.7b07532.

(2) Bolovinos, A.; Tsekeris, P.; Philis, J.; Pantos, E.; Andritsopoulos, G. Absolute Vacuum Ultraviolet Absorption Spectra of Some Gaseous Azabenzenes. *J. Mol. Spectrosc.* **1984**, *103* (2), 240–256. https://doi.org/10.1016/0022-2852(84)90051-1.

(3) Sala, M.; Guérin, S.; Gatti, F. Quantum Dynamics of the Photostability of Pyrazine. *Phys. Chem. Chem. Phys.* **2015**, *17* (44), 29518–29530. https://doi.org/10.1039/C5CP04605J.

(4) Tsuru, S.; Vidal, M. L.; Pápai, M.; Krylov, A. I.; Møller, K. B.; Coriani, S. Time-Resolved near-Edge X-Ray Absorption Fine Structure of Pyrazine from Electronic Structure and Nuclear Wave Packet Dynamics Simulations. *J. Chem. Phys.* **2019**, *151* (12), 124114. https://doi.org/10.1063/1.5115154.

(5) Sala, M.; Lasorne, B.; Gatti, F.; Guérin, S. The Role of the Low-Lying Dark $n\pi^*$ States in the Photophysics of Pyrazine: A Quantum Dynamics Study. *Phys. Chem. Chem. Phys.* **2014**, *16* (30), 15957–15967. https://doi.org/10.1039/C4CP02165G.

(6) Stert, V.; Farmanara, P.; Radloff, W. Electron Configuration Changes in Excited Pyrazine Molecules Analyzed by Femtosecond Time-Resolved Photoelectron Spectroscopy. *J. Chem. Phys.* **2000**, *112* (10), 4460–4464. https://doi.org/10.1063/1.481008.

(7) Horio, T.; Spesyvtsev, R.; Nagashima, K.; Ingle, R. A.; Suzuki, Y.; Suzuki, T. Full Observation of Ultrafast Cascaded Radiationless Transitions from $S_2(\pi\pi^*)$ State of Pyrazine Using Vacuum Ultraviolet Photoelectron Imaging. *J. Chem. Phys.* **2016**, *145* (4), 044306. https://doi.org/10.1063/1.4955296.

(8) Mai, S.; Marquetand, P.; González, L. Nonadiabatic Dynamics: The SHARC Approach. *WIREs Comput. Mol. Sci.* **2018**, *8* (6), e1370. https://doi.org/10.1002/wcms.1370.

(9) Barbatti, M.; Granucci, G.; Persico, M.; Ruckenbauer, M.; Vazdar, M.; Eckert-Maksić, M.; Lischka, H. The On-the-Fly Surface-Hopping Program System Newton-X: Application to Ab Initio Simulation of the Nonadiabatic Photodynamics of Benchmark Systems. *J. Photochem. Photobiol. Chem.* **2007**, *190* (2), 228–240. https://doi.org/10.1016/j.jphotochem.2006.12.008.

(10) Stener, M.; Decleva, P.; Holland, D. M. P.; Shaw, D. A. A Study of the Valence Shell Electronic States of Pyrimidine and Pyrazine by Photoabsorption Spectroscopy and Time-Dependent Density Functional Theory Calculations. *J. Phys. B At. Mol. Opt. Phys.* **2011**, *44* (7), 075203. https://doi.org/10.1088/0953-4075/44/7/075203.

(11) Penfold, T. J.; Worth, G. A. A Model Hamiltonian to Simulate the Complex Photochemistry of Benzene II. *J. Chem. Phys.* **2009**, *131* (6), 064303. https://doi.org/10.1063/1.3197555.
(12) Guo, J.-L.; Liu, C.; Xie, B.-B.; Zhao, Y.-Y.; Pei, K.-M.; Wang, H.-G.; Zheng, X.; Ai, Y.-J.; Chen, X.-B.; Fang, W.-H.; Yeung, C. S. Vibronic Coupling and Excited-State Reaction Dynamics of Pyrazine in 1 $^1B_{2u}$ ($^1\pi\pi^*$) State by Resonance Raman Spectroscopy and CASSCF Calculation. *J. Raman Spectrosc.* **2012**, *43* (10), 1477–1486. https://doi.org/10.1002/jrs.4074.
(13) Piancastelli, M. N.; Keller, P. R.; Taylor, J. W. Angular Distribution Parameter as a Function of Photon Energy for Some Mono- and Diazabenzenes and Its Use for Orbital Assignment. *J. Am. Chem. Soc.* **1983**, *105* (13), 4235–4239. https://doi.org/10.1021/ja00351a019.
(14) Epshtein, M.; Scutelnic, V.; Yang, Z.; Xue, T.; Vidal, M. L.; Krylov, A. I.; Coriani, S.; Leone, S. R. Table-Top X-Ray Spectroscopy of Benzene Radical Cation. *J. Phys. Chem. A* **2020**, *124* (46), 9524–9531. https://doi.org/10.1021/acs.jpca.0c08736.
